# Observations on Some Affections of the Fingers and Toes

**Published:** 1831-01-01

**Authors:** 


### Observations on some of the Affections of the Fingers and Toes, attended with Fungous Growths. By Francis Rynd, A.B., &c. &c.

To all who are engaged in the practice of their profession, the affections of the fingers and toes will not be devoid of interest, though speculative philosophers may deem them of very subordinate character. They have attracted much attention from the earliest ages in which medicine was cultivated, and yet they are not seldom an opprobrium to the art of surgery at present. The papers of Mr. Wardrop and M. Royer Collard are acknowledged by Dr. Rynd to be good, yet he thinks that the results of his own experience may not be unprofitable additions to the observations of those writers.

The surgeon should always remember well the nature of that membrane by which the nail is secreted, and bear in mind that it is reflected around, or rather behind the root of the nail, at the distance of two or three lines from its posterior border. It is exquisitely sensible, and much disposed to throw out fungous growths.

"I believe the membrane or matrix of the nail exhibits three forms of fungoid growth, similar in their appearances, and the distress they occasion, differing in their situations and the causes by which they are produced. One of these is where the matrix is the original seat of the disease; it occurs most frequently idiopathically, or at least no satisfactory cause can be assigned for its production, but occasionally it seems to arise from some external violence, such as a blow or bruise. The second is that which owes its origin to pressure of the nail on the membrane, or what is termed the nail growing into the flesh. And the third *is a fungous growth of the matrix, symptomatic of, and apparently occasioned by, the existence of an abscess underneath it.* That the two first of these may eventually become the same, or that the constant pressure of the edge of the nail on this most sensible structure will involve it altogether in one mass of disease, cannot be controverted, and then the same treatment will be applicable to both: but if the third or symptomatic species happens to be mistaken for either of them, and the surgeon's attention be directed to the fungus instead of to the abscess, of which it is an indication, months of misery will be inflicted on the patient, who only recovers when the abscess bursts or is opened.

That disease, which for the sake of arrangement I have termed idiopathic, and which is perhaps the true *onychia maligna*, commences usually at the root of the nail, it may be with a small collection of matter like the cutaneous or superficial paronychia. This bursts, and the posterior edge of the nail is seen detached, while a small pale-coloured and exquisitely painful fungus projects behind it; as the nail is thrown off, the fungus increases, becomes red, and if pressed upon by the nail, the pain is excruciating: sooner or later the entire nail is thrown off, but it does not leave a fungous growth regularly occupying its former situation; the matrix has been secreting its proper material of which the nail is composed, but it has done so irregularly, unhealthily, and in patches, and the irritation and pressure occasioned by those small portions of the nail which usually adhere to the surface by one edge only, produce deep and formidable ulcerations. The extremity of the toe or finger (and the great toe is very frequently enlarged) now appears to be expanded in breadth, with a large ulcer occupying a larger space than that of the former nail. Its surface is irregular, partly fungoid, partly excavated; on it are observed small portions of nail firmly adherent, and which cause great pain if pulled or pressed upon. The ulcer bleeds often, and usually two or three small clots are seen upon its surface: its discharge is not often profuse, but it is glutinous, and the dressings adhere to it. The margin of the sore is elevated, of a dark red colour, but it does not spread, and having once attained to a given size, something larger than that of the former nail, its progress is checked, and it remains for months in nearly the same condition, occasioning the greatest pain, and rendering the hand or foot, as the case may be, nearly useless."

The circumstances that produce this malignant tendency in the matrix of the nail have never been explained, and Mr. Rynd looks on the fungus as an idiopathic morbid alteration of structure, requiring the complete removal of the texture from which it grows. Evulsion of the diseased or imperfectly formed portions of nail is one of the most ancient methods; it does not remove the source of the fungus. Various caustic applications to the latter have been used, but although continued for months they would not effect a cure, as they do not reach that part of the matrix belonging to the root of the nail and protected by the integument. The removal of the matrix by the knife, recently attributed to Dupuytren was practised in the Dublin hospitals for years before the French surgeon's practice was made public. The operation consists in making a deep incision down to the bone, from three to four lines behind the posterior margin of the sore, carrying the incision round it, and dissecting out the entire surface. The wound is dressed simply and is usually healed in the course of sixteen or eighteen days. The success of the operation depends on the *entire* surface being removed.

### II. Growing of the Nail into the Flesh.

Mr. Rynd is inclined to doubt whether this depends so much on the pressure of tight shoes as is commonly supposed. Be this as it may, when the pressure and irritation of the nail give rise to a fungus from the ulcerated fissure at its side, the matrix is not universally diseased, as in the onychia maligna.

"From an examination of a number of cases of this description, many of which I was enabled to see through the kindness of some professional friends, who were aware of the interest I had taken in them, I have been induced to believe that a very considerable variety exists as to the nature of these affections. In some it consists of a thickened condition of the nail, together with its taking a wrong direction in some part, and growing fairly down into the soft parts. This thickening of the nail is very analogous to the formation of corns, rarely produces much greater inconvenience, is seldom accompanied by fungous growths, and is relieved in a manner similar to that by which corns are relieved, by steeping the foot in warm water until the nail is completely softened, scraping down the nail with the edge of a bit of glass, and freeing the toe from the pressure that had stimulated the matrix to too active a secretion of the material of the nail and thus produced the disease. In other forms of this affection the nail is not thickened, on the contrary, its edge sometimes is thinner where it is detached from the subjacent membrane which is pushed against it, becomes fungoid, and thus produces the disease within itself. In this affection the toe positively becomes altered in shape; it is contracted and turned slightly upwards at the side whereon the nail seems to press, and it is this contraction which forces the soft parts against the edge of the nail."

The most common operation for this affection has been dividing the nail longitudinally in its whole extent, and then tearing off the offending portion or portions. This is acknowledged to be horribly painful, and Mr. Rynd avers that it is generally inefficacious, the disease reappearing as the new nail is formed. In short he proposes the measure which is adopted in onychia maligna, the removal of the nail, matrix, and all by the knife. This has been done with uniform success by a particular friend of Mr. Rynd, one of the surgeons of the Meath Hospital.

### III. Fungus from Abscess existing within the Finger.

Mr. Rynd is not aware that this affection has hitherto been noticed by any surgical writer. An abscess forms within the finger; the membrane or matrix secreting the nail becomes fungous, and remains so till the abscess either bursts or is opened; and till that occurs there is the most acute agony, which then disappears spontaneously. Of this we are presented with two examples. We shall reverse their order and give the last first.

*Case* 1. An eminent medical practitioner in Dublin suffered from inflammation of the thumb, produced by some trifling injury. In a short time a crack or fissure, from which flowed a serous discharge, appeared anteriorly between the nail and the flesh, and from this sprouted a fungus which gave much pain. A deep incision was made through the fungous growth, an abscess opened, and the patient was well in eight and forty hours.

*Case* 2. A medical gentleman, æt. 38, corpulent and rather indolent, who had previously enjoyed good health, with the exception of occasional attacks of synanche tonsillaris, was much employed in anatomical pursuits during the Winter season of 1827-28. He had been married twelve years, and had nine healthy children, and could not possibly have contracted any syphilitic taint. In June, 1828, he stuck the point of a *new pin* into the extremity of his left thumb, but not under the nail. We should state that three weeks previously he had received a slight cut on the thumb, whilst examining a patient that had died of diseased hip-joint, but this wound had healed speedily and well. In the course of a day or two after the prick from the pin, the thumb became painful, slightly swollen, and red, and its appearance was that of a slight attack of gout. So it remained for three weeks when a crack appeared under the anterior edge of the nail, followed by a trifling oozing of serum. Immediately a small fungus sprang up, pressed against the nail, and occasioned intense suffering. A portion of the nail was removed, but still the matrix, where exposed, became fungous, till nearly one half of it was in that condition. One gentleman suspected the existence of abscess, an incision was made, but no matter was found; this was repeated in the course of two days with the same want of success. The patient's sufferings were extreme, his health declined, and he became weak and wasted. After lying in this state for two months, pressure on the fungus was tried with sheet lead, and during the night he experienced sudden relief; the abscess had burst and the fungus disappeared in forty-eight hours, but an unhealthy irritable ulcer remained, which took a longer time to heal. But the curious part of the case is to be told, which we cannot do better than in Mr. Rynd's own words. The sequel is in fact extremely curious.

"About three weeks after the first appearance of the fungus, the lymphatics of the fore-arm became inflamed; the inflammation extended along their course to the axilla; sixty leeches were applied and the inflammation was subdued. About a month afterwards an enlarged gland appeared in the axilla, which was also resolved by the application of leeches.

On the 27th of September the abscess burst, and on the 29th he went to the country emaciated and debilitated to an extent not to be explained by the effects of so small a local disease. On the 10th of October his throat felt sore, with some trifling difficulty of swallowing; he returned to Dublin, his throat was examined, and no appearance of disease was discovered except a very slight blush of inflammation, which soon passed away. Towards the latter end of October an eruption of scarlet patches came out on his forehead, fore-arms, and slightly on the shoulders and inside of the thighs. Sarsaparilla and the nitro-muriatic acid were now prescribed, and taken in considerable quantity. In this state he remained until the end of December, his throat being on one day free from uneasiness, and perhaps on the next extremely sore, the eruption sometimes disappearing entirely, sometimes shewing faintly under the cuticle like fading measles, and sometimes of a bright red scarlet colour. He continued taking the sarsaparilla up to this period, with warm baths twice a week, and his general health had been improving since the sore on the thumb had healed. He got weary of taking medicine, and now discontinued it, and trusted to a nutritive diet and the use of port wine.

Early in January, 1829, an anomalous kind of eruption appeared on his scrotum, and produced great annoyance it was so intolerably itchy, but it scaled off and disappeared in the course of a week without the adoption of any medical treatment whatever. On the 15th a tender tumefaction arising from periostitis was discovered on the left tibia, on the 19th it disappeared. On the 2nd of February an eruption similar to what had before appeared on the scrotum re-appeared; it also went away without the use of medicine. During the months of March, April, and May, his throat could never be considered in a state of health, and exhibited so much variety of symptoms, both as to appearance and pain, as rendered the case inexplicable as it was curious; sometimes when there was no trace of inflammation visible the pain would be excessive. An erythematous redness was, on the other hand, often suffused over the soft palate, and occasionally an appearance resembling abrasion of the surface, while there were no indications of exacerbation of pain. Sometimes whilst speaking his voice would suddenly fail, and a paroxysm of cough interrupt his articulation; the cough was hard and unattended by expectoration; he would often commence eating, suffering under extreme torture in every attempt to swallow, and after a few moments he would perform deglutition without the slightest pain. In a word, these symptoms came and went alternately without any assistance being sought from medicine. But this uncertain and precarious state of health could not be borne any longer, and he determined to try the effects of mercury, though contrary to the advice of his friends, and took two grains of calomel daily for four days, when he was seized with a cough, accompanied with some hæmoptoe. The mercury was discontinued immediately, and never resumed: his mouth was made slightly sore by this quantity, his gums became spongy, and his breath foul. All the symptoms gradually and slowly declined, and he is now occasionally visited by the following. Wandering pain in the hip-joint of the right side, and weakness which prevents exercise on foot to any considerable extent. Some few pale and almost indistinct stains of eruption on his forehead after exercise. Some broad, flat patches of eruption of a scarlet colour, but not always of the same intensity, on the back of the hands; during the last winter the skin on the palms of his hands was hard and horny, and the natural lines appeared like fissures; this has now disappeared. His throat never has been sore since he took the calomel."

The secondary symptoms in this case, and their order of occurrence are remarkable and deserving of attention. We look on Mr. Rynd's paper as an acquisition in this department of surgery.

